# Dr. Paul Tessier

**Published:** 2008

**Authors:** Surajit Bhattacharya

**Affiliations:** Consultant Plastic Surgeon, In-coming Editor, Indian Journal of Plastic Surgery, Lucknow, India Email: surajitbh@yahoo.co.in

Dr. Paul Tessier's death comes as a rude shock to the Plastic Surgery community all around the world. Dr. Tessier, who was residing in Boulogne, France, was born in Brittany and attended medical school at the Ecole de Medicine in Nantes, France, and received his Doctor of Medicine from the Faculte de Medecine de Paris in 1943. From 1939 until 1941, Dr. Tessier was in military service, the last year of which he spent as a prisoner of war in a German military hospital.

**Figure:**
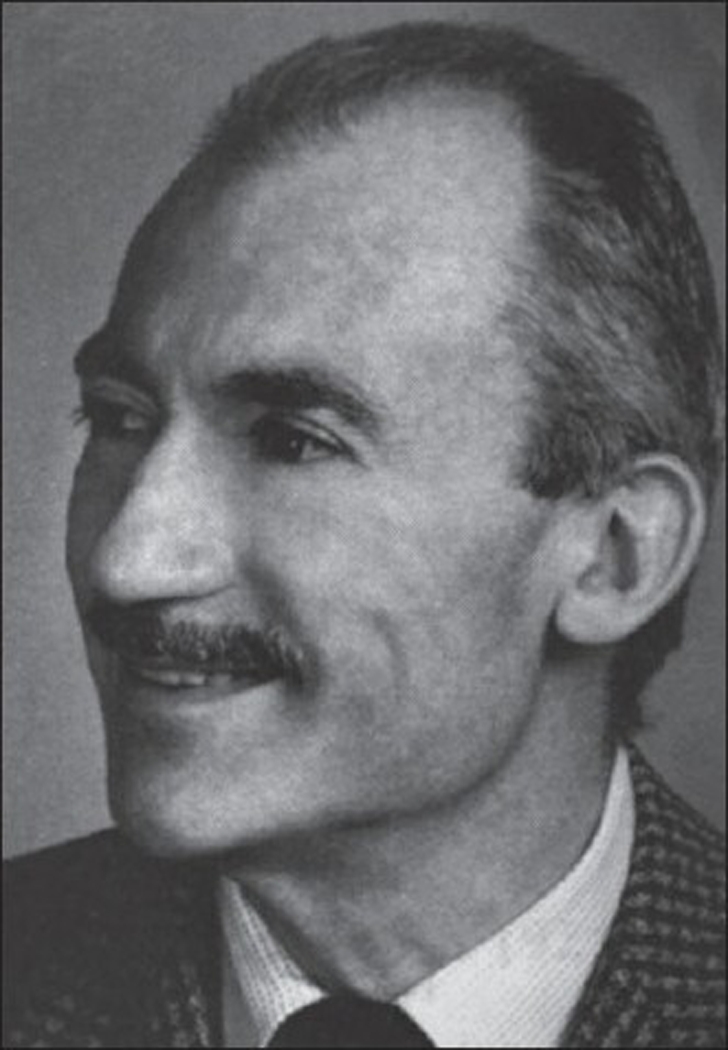
Dr. Paul Tessier

In 1942, Dr. Tessier interned with a general surgeon who operated on cleft lip and Dupuytren's contracture. He continued his work in the surgical profession by going to Paris and joining the pediatric surgery service at Hospital St. Joseph in 1944. Later that year, while Paris was still under occupation by Germany, Dr. Tessier became an assistant to the Center of Maxillo-Facial Surgery of the Military Region of Paris in Hospital Puteaux where he remained until 1946. In 1947, the hospital's administration was transferred to Hospital Foch (Paris), and 2 years later, Dr. Tessier became involved in plastic surgery and burn care. In 1949, he returned to Nantes where he became a surgical consultant in ophthalmology.

In the mid 1950's, Dr. Tessier began innovative work on finding a way to perform osteotomies to correct congenital midfacial retrusion without the relapse which plagued the performance of the procedure years before by one of his mentors, Sir Harold Gillies. In 1958, he began his initial clinical practice of the overhauled procedure. In discovering the need for bone grafts to fill gaps in the bone that could potentially jeopardize the adjustments made during the procedure, Dr. Tessier started the surgical specialty of cranofacial surgery.

Within 3 years, Dr. Tessier had improved on his initial work by providing exposure for much of the procedure through a coronal approach, he had begun to section the arch rather than the body of the zygoma, and he placed bone grafts in the pterygomaxillary gap, all of which gave added stability to the midface segment without requiring an external head frame. In 1967, he performed a number of demonstration procedures for his peers and asked for their vote on whether this new specialty had a place in the practice of surgery. The positive response launched craniofacial surgery into the profession.

During the late 1960's and the 1970's, Dr. Tessier developed all of the procedures that are currently used in performing craniofacial surgery: transcranial and subcranial correction of orbital dystopias such as orbital hypertelorism, correction of the facial deformity of Teacher Collins Franceschetti syndrome, and correction of oro-ocular clefts. Also, in the 1970's, Dr. Tessier began traveling to the U.S. in order to demonstrate the procedures in cities such as Philadelphia, Chicago, Houston, Boston, and Norfolk.

Dr. Tessier started to improve surgical techniques to correct craniofacial deformations in mid-1950s. Throughout the 1960s and 1970s, he developed the following methods:

- Using autogeneous bone grafts instead of silicone or acrylic to modify skull and facial contours.
- Transcranial and subcranial correction of orbital hypertelorism.
- Techniques for correcting Treacher Collins syndrome.
- Correction of oro-ocular clefts.

The impact of Dr. Tessier's work has affected many surgical specialties, including plastic surgery, otorhinolaryngology, ophthalmology, neurosurgery, trauma surgery, and oral and maxillofacial surgery. Many of his techniques have found significant places in the performance of plastic surgery, where Dr. Tessier's methods of autogenous bone grafts have often been seen as an improvement over the traditional use of silicone or acrylic. He also coined the term SMAS and created the subperiosteal facelift, or masklift. Dr. Tessier's work is so far reaching that virtually everyone performing procedures of the craniofacial specialty were either trained by Dr. Tessier himself, or by surgeons he trained.

Because of his work, Dr. Tessier was a founding member of the International Society of Craniofacial Surgery and the European Association of Maxillo-Facial Surgeons, and had been honored many times, including receiving honorary memberships in the American College of Surgeons, the Royal College of Surgeons (London), and the American Society of Plastic Surgeons. In addition, he has been given an honorary degree from Lund University in Sweden.

Paul Tessier, MD, FACS (Honorary), was the recipient of the Jacobson Innovation Award of the American College of Surgeons for the year 2000. The purpose of this award is to honor living surgeons, or surgical teams, who have been innovators of a new development or technique in any field of surgery. Dr. Tessier won this award for his original thought combined with the first presentation of work that has led to a milestone in the advancement of surgical care. The fact that Cranio-facial Surgery is so popular the world over is an ample testimony to the greatness of this colossal man of surgical sciences.

May his soul rest in peace.

